# Transfer learning for ECG classification

**DOI:** 10.1038/s41598-021-84374-8

**Published:** 2021-03-04

**Authors:** Kuba Weimann, Tim O. F. Conrad

**Affiliations:** 1 Department of Visual and Data-Centric Computing, Zuse Institute Berlin, Takustrasse 7, 14195 Berlin, Germany; 2 Department of Mathematics, Free University of Berlin, Arnimallee 6, 14195 Berlin, Germany

**Keywords:** Computational biology and bioinformatics, Machine learning

## Abstract

Remote monitoring devices, which can be worn or implanted, have enabled a more effective healthcare for patients with periodic heart arrhythmia due to their ability to constantly monitor heart activity. However, these devices record considerable amounts of electrocardiogram (ECG) data that needs to be interpreted by physicians. Therefore, there is a growing need to develop reliable methods for automatic ECG interpretation to assist the physicians. Here, we use deep convolutional neural networks (CNN) to classify raw ECG recordings. However, training CNNs for ECG classification often requires a large number of annotated samples, which are expensive to acquire. In this work, we tackle this problem by using *transfer learning*. First, we pretrain CNNs on the largest public data set of continuous raw ECG signals. Next, we finetune the networks on a small data set for classification of Atrial Fibrillation, which is the most common heart arrhythmia. We show that pretraining improves the performance of CNNs on the target task by up to $6.57\%$, effectively reducing the number of annotations required to achieve the same performance as CNNs that are not pretrained. We investigate both supervised as well as unsupervised pretraining approaches, which we believe will increase in relevance, since they do not rely on the expensive ECG annotations. The code is available on GitHub at https://github.com/kweimann/ecg-transfer-learning.

## Introduction

Remote monitoring devices, which can be worn or implanted, have enabled a more effective healthcare for patients with periodic heart arrhythmia due to their ability to constantly monitor heart activity. At the same time, these devices record large amounts of electrocardiogram (ECG) data that needs to be interpreted. This task falls on physicians, nurses and other medical workers. The additional workload further contributes to the fatigue regularly experienced by the medical staff at work, which increases the chances of medical errors^1,2^. Additionally, a significant portion of the received ECG recordings are often false alarms, since the remote monitoring devices are very sensitive to abnormalities in the ECG, which prevents them from missing major cardiovascular events. Therefore, there is a growing need to assist the physicians with the interpretation of ECG recordings.

Worldwide, millions of ECG recordings are collected annually, majority of them automatically analyzed and interpreted by computers^3^. This imposes the requirement on the ECG interpretation methods to not only be fast and accurate but also patient and device independent. The widespread digitization of ECG data coupled with the development of deep learning methods, which can process large amounts of raw data, has introduced new possibilities for improving the automated ECG interpretation. Indeed, deep neural networks (DNN) have recently achieved cardiologist-level classification performance^4^ when trained on a large (n = 91,232) data set of raw ECG recordings. However, available ECG data sets are often much smaller, which makes it difficult to achieve a desirable performance level. In this work, we focus on the case where the data set used for training a classifier is small.

The properties of ECG data captured by remote monitoring devices pose several challenges for training DNNs to classify ECG data. In particular, the main challenges are: (1) a drastic class imbalance caused by the rare occurrence of some cardiovascular events; (2) a low signal quality, e.g. reflected by a low sampling frequency, single ECG lead and noisiness; and (3) a small number of annotations owing to the substantial costs of employing experts to manually label the ECG recordings. In this work, we focus on the third challenge. In supervised learning, the performance of a classifier depends on the size of data set. Thus, we investigate whether including ECG data from other sources into the training process improves the classification performance, despite the differences in the properties of ECG signals (e.g. due to different recording devices) and labels (e.g. due to a different purpose of data collection). To that end, we include new ECG data with and without labels. Although in both cases the size of training data grows larger, a greater increase is expected from using unlabeled data sets because they are collected without manual intervention.

There are different approaches to improving classifiers when manual labeling becomes too expensive. For instance, *active learning* aims to guide the labeling process by ranking the unlabeled examples according to some criterion that selects the most useful examples for improving the model. These examples are given to the experts for labeling and after that the model is retrained. Thus, the number of training samples necessary to maintain high discriminative capabilities is minimized. Nevertheless, the need for manual labeling persists, albeit to a lesser extent. At the same time, *semi-supervised learning* combines small amounts of labeled data with a large amount of unlabeled data to improve the learning accuracy, e.g. by learning unsupervised representations of the data^5^ or feature extractors that improve the downstream (target) task. Finally, *transfer learning* focuses on gathering knowledge by solving one problem and applying it to a related problem in the same domain. In computer vision, most state-of-the-art classification algorithms rely on supervised pretraining that roughly follows the same procedure: first pretrain a convolutional neural network on a large labeled data set (e.g. ImageNet^6^), then finetune the network on a smaller target data set.

**Figure 1:**
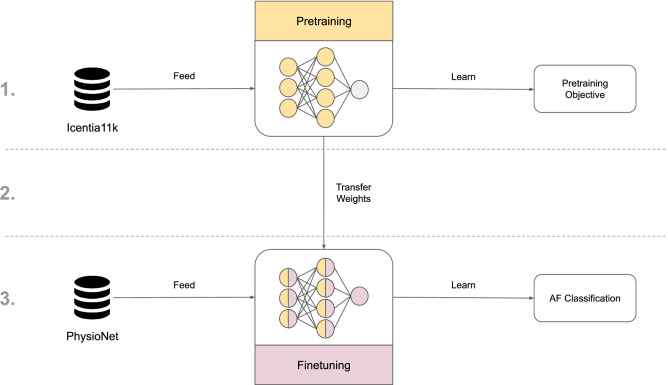
Visualization of transfer learning in this work. The process is divided into 3 steps: (1) deep convolutional neural network (CNN) is pretrained on the Icentia11K^5^ data set for a selected pretraining objective, e.g. classification of heart rate; (2) the pretrained weights are used as initial weights of a new CNN; (3) this CNN is finetuned on the PhysioNet/CinC Challenge 2017^7, 8^ data set to classify Atrial Fibrillation (AF).

**Figure 2:**
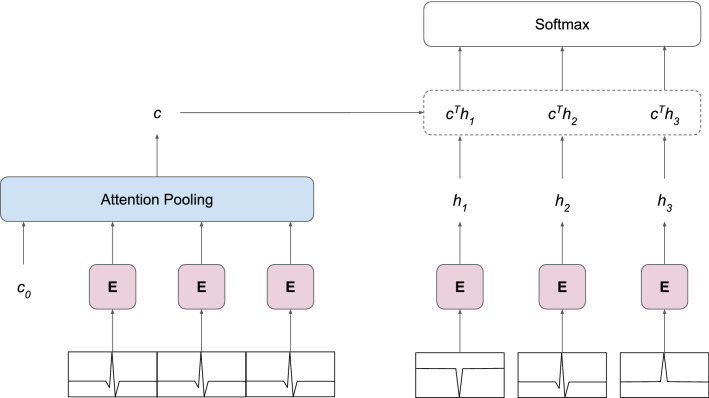
Architecture of the future prediction framework. The model predicts the correct future frame among negative samples (right) based on the present frames (left). Encoder **E** extracts features from ECG frames, producing a feature vector for each frame. Attention pooling summarizes feature vectors into a single context vector *c* describing the present. A dot product between *c* and frame encodings $h_i$ gives the similarity between the context and future frames. The entire model is trained end-to-end with gradients backpropagated from the cross-entropy loss of classifying the future frame correctly.

**Figure 3:**
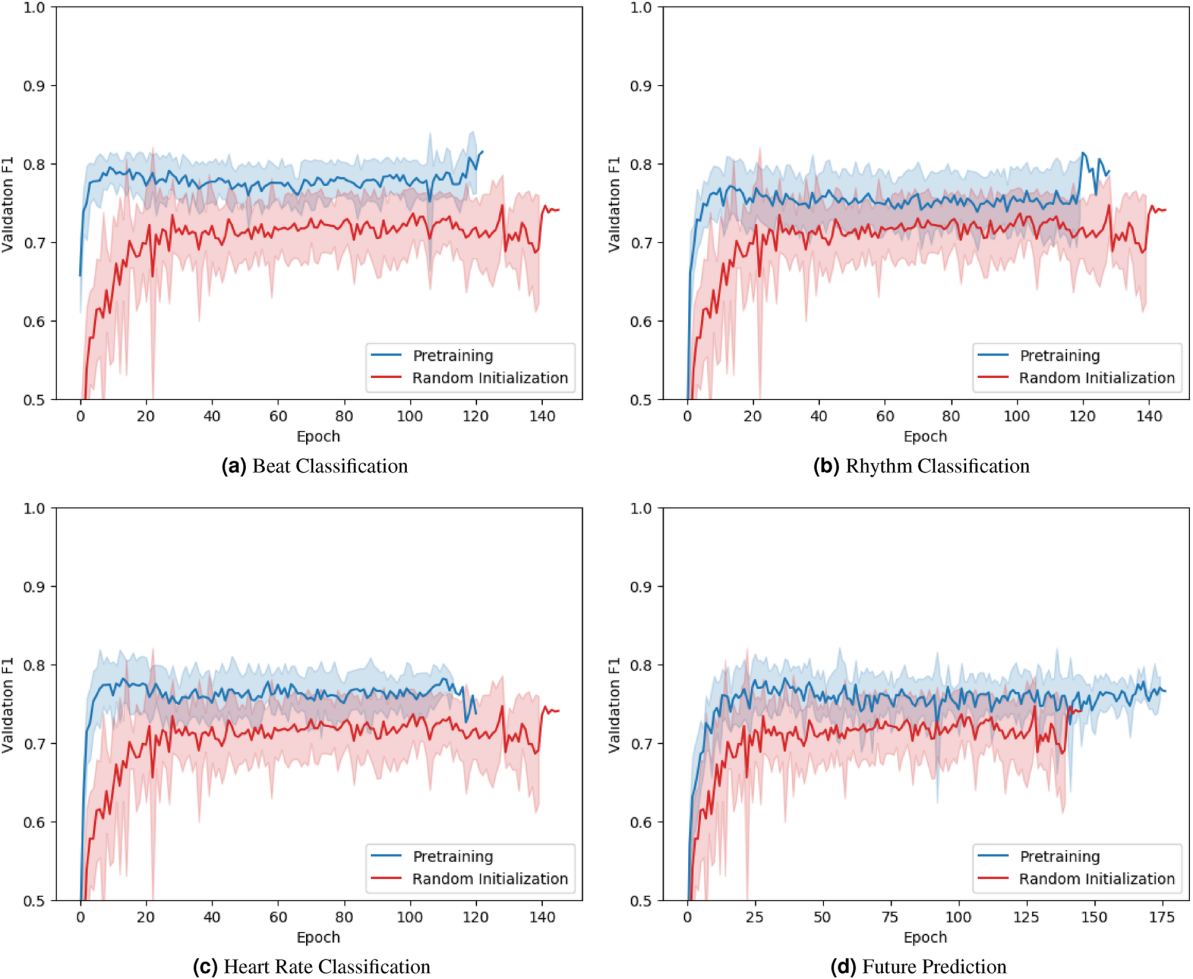
Average macro $F_1$ on the validation set during finetuning. At each epoch, the average validation score of a selected pretraining method (blue) and random weight initialization (red) is reported. Pretraining improves validation performance on the downstream task and accelerates the training.

In this work, we use transfer learning to improve ECG classifiers (Fig. 1). First, we pretrain deep convolutional neural networks (CNN) on the Icentia11K^5^ data set. To that end, we define several pretraining tasks that utilize either labeled or unlabeled data. Next, we finetune the pretrained CNNs on the PhysioNet/CinC 2017 data set^7,8^ to classify Atrial Fibrillation (AF). AF is the most common heart arrhythmia^7^ characterized by an irregular heart rhythm that is caused by a chaotic propagation of electrical impulses in the atria. AF can lead to serious complications such as heart disease, heart failure, or blood clots that can travel to the brain and cause a stroke.

Our main contribution is a successful large-scale pretraining of CNNs on the largest public ECG data set to date. By pretraining CNNs, we improve their performance on the target task and effectively reduce the number of expensive annotations required to achieve the same performance level as CNNs that are not pretrained. Our pretraining methods are robust to changes in the properties of ECG signals and can be applied to different models and data sets. Notably, we show how a CNN, which was pretrained on single lead ECG data, can be finetuned on 12 lead ECG data. Further, we show how contrastive pretraining, which is an unsupervised representation learning technique, can improve the performance of CNNs on the target task. Contrastive learning has been extensively researched in computer vision, leading to many state-of-the-art results in transfer learning^9,10^. However, applications to ECG data are not common. Finally, our pretraining tasks include heart rate classification, whose labels require no manual intervention to create. To the best of our knowledge, this task has not been previously used for pretraining.

## Related work

Automatic ECG interpretation using deep learning has attracted a lot of attention in the recent years. A good overview of the current state of deep learning methods for processing ECG data can be found in Faust et al.^11^ and Hong et al.^12^ Here, we only discuss the publications that are most relevant to our work. Furthermore, we briefly compare these publications with our work.

### ECG classification

Most ECG classification methods for disease detection can be categorized as either heartbeat^13–15^ or heart arrhythmia classification^4,16–18^ based on some form of ECG signal as the input to a neural network. The input is usually a 1-dimensional raw ECG signal^4,16–18^ and less commonly a 2d image of the signal^14^ (e.g. by plotting a grayscale image of the signal), or a spectrogram of the signal^19,20^. Here, we follow the trend of training 1d convolutional neural networks (CNN), in particular residual networks^21,22^, on raw ECG signals for heart arrhythmia classification. In addition to that, we employ an attention model based on the Transformer architecture^23^ for summarizing features from short ECG frames in one of the pretraining tasks. Although attention models were originally used in natural language processing^24,25^, they also found application in the domain of ECG interpretation^19^. Furthermore, in this work we focus on ECG data collected by remote monitoring devices that are typically less informative than clinical devices, e.g. due to a smaller number of leads. Nonetheless, remote monitoring devices, such as wristbands, are becoming increasingly common, facilitating collection of large ECG databases. As a consequence, a lot of work has been devoted to automatic interpretation of this kind of data. However, existing large ECG databases remain mostly inaccessible to the general public, thus a lot of research is done using relatively small public data sets, for instance PhysioNet/CinC Challenge 2017^7,8^ data set, which is used for AF classification^4,20,26^. In this work, we measure the performance of our pretraining methods on the PhysioNet/CinC Challenge 2017 data set.

### Transfer learning

Although most studies focus on classification of arrhythmia on a data set annotated for that purpose, there are some publications that use transfer learning to share knowledge between related tasks, effectively utilizing the commonalities between different ECG conditions. For instance, Kachuee et al.^27^ presented a method for ECG heartbeat classification based on transferable representations using 1-dimensional residual networks. Here, we deal with ECG frames much longer than a heartbeat, which we use to classify heart arrhythmia. More recently, Strodthoff et al.^28^ used transfer learning on public ECG data sets to classify heart arrhythmia. Similar to their work, we finetune the pretrained networks to classify heart arrhythmia, however, we use a much larger upstream data set for pretraining and investigate several pretraining tasks. There are also studies^29,30^ of transfer learning from 2-dimensional deep CNN features trained on ImageNet^6^, a data set often used for pretraining computer vision models. In contrast to these studies, we focus exclusively on transferable ECG representations, not transferable image representations.

### Representation learning

Machine learning research in healthcare is often limited by the lack of large annotated data sets. The performance of existing solutions for supervised tasks can be improved by learning unsupervised representations as part of a multi-stage training process. Using such representations may also help discriminative models to generalize better to unseen data, which often proves to be a serious issue. To that end, there are several studies which explore encoder-decoder architectures in order to learn unsupervised representations. In particular, Rahhal et al.^31^ and Xia et al.^32^ train stacked denoising autoencoders to reconstruct ECG heartbeats. Tan et al.^5^ reconstruct slightly longer ECG frames using a simple autoencoder, and Rajan et al.^33^ train an encoder-decoder network composed of RNNs (also referred to as *Seq2Seq* model) to reconstruct missing channels from a short ECG frame. In all of the above examples, the latent space learned by the generative model is exploited by training a classifier on top of it. However, generative models taught to reconstruct the input often fail to extract context information which may be useful for the downstream (target) task^34^. Therefore, in our work, we utilize the Contrastive Predictive Coding approach^34^ that learns to infer global structure in the signal, rather than only model complex local relationships.

## Method: transfer learning for ECG classification

Transfer learning applies knowledge obtained by solving one problem to a different but related problem. The general procedure for transfer learning is to first *pretrain* a deep neural network (DNN) on a large data set (i.e. upstream data set), then *finetune* the DNN on a much smaller target data set (i.e. downstream data set). Pretraining provides us with a way to find good initial weights that improve the learning of the target task thanks to some already acquired knowledge in the task domain. In particular, we hope that the pretrained DNNs will generalize better to unseen data after encountering a wide variety of ECG signals during pretraining.

In this work, we first pretrain deep convolutional neural networks (CNN) on the Icentia11K^5^ data set, which is the largest public ECG data set to date. To that end, we have defined several pretraining tasks that we will investigate. Next, we finetune the pretrained CNNs for classification of Atrial Fibrillation on a data set released as part of the PhysioNet Computing in Cardiology Challenge 2017^7,8^, which we refer to as the PhysioNet/CinC Challenge 2017 data set. In the following subsections, we describe in detail our approach to transfer learning for ECG data and introduce the data sets that the CNNs are trained on.

### Pretraining

We pretrain CNNs on Icentia11K, which contains ECG data from 11,000 patients. The average patient is about 60 years old and exhibits some form of heart arrhythmia. As a consequence, the data set is not a representative sample of the population. Each patient wore a CardioSTAT device for a period of up to two weeks. The recorded data is a raw ECG signal sampled at 250 Hz in a modified lead I position. Beside recording the ECG signal, the device performs automatic beat detection. The beat labels are verified by a specialist who also labels the heart rhythm in a full disclosure analysis, i.e. whole recording is examined. Both beat and rhythm labels are assigned to positions in the signal at irregular intervals. The data set is a collection of up to 50 segments of continuous ECG signal sampled from every patient. We reserve segments from 5% of patients for validation, and use the remaining segments for training. To put things into perspective, the data set contains over 630,000 h of ECG signal with over 2,700,000,000 labeled beats.

During pretraining, we collect mini-batches by sampling short ECG frames from randomly chosen patients. We consider an ECG frame to be a fragment of the continuous ECG signal that is less than a minute long. We standardize each frame using mean and standard deviation computed over the entire data set. On average, we sample 4096 ECG frames per patient, which amounts to 42.8 million training samples over the course of pretraining. Every couple thousand training steps, we save the weights of the CNN in a checkpoint. Once the pretraining is finished, we revert the model to the checkpoint, at which the model had the highest validation accuracy. Below, we describe how we design the tasks for pretraining a CNN on the Icentia11K data set.

### Beat classification

CNN predicts the presence of an abnormal beat in a short ECG frame. For each frame, we look for the occurrence of either premature atrial contractions (PAC), premature ventricular contractions (PVC), or aberration. If no abnormality is found, the frame is labeled as normal beat. In rare cases of multiple abnormal beats within a single frame, we select the most frequent beat type for the label.

### Rhythm classification

CNN predicts the heart rhythm from a short ECG frame. For each frame, we look at the duration of every rhythm to determine the label. Specifically, we pick the longest of the rhythms, while prioritizing Atrial Fibrillation (AFib) and Atrial Flutter (AFlut). This means that we first select the longer among AFib and AFlut if they are present, otherwise the longest of the remaining rhythms, i.e. normal sinus rhythm or noise.

### Heart rate classification

CNN predicts the heart rate from a short ECG frame. In contrast to beat and rhythm classification, where labels are in part created by specialists, the labels for this task are generated automatically, i.e. without human intervention. For each frame, we first find the indices of heartbeats (this can be done using a QRS detection algorithm^35^) and estimate the number of beats per minute (BPM) based on the interbeat intervals. Next, we assign a class based on BPM: Bradycardia (< 60 BPM), Tachycardia (>100 BPM), Normal (60–100 BPM) and Noise in case of a failure to detect any heartbeats. Additionally, when generating the label, we always temporarily extend the size of the frame by 1 s at both ends. We consider this as a way to improve the quality of labels, especially when the frames are very short, which can make the labeling process more susceptible to variations in the heart rate. Note that since the labels are generated based on an ECG frame that is longer than the actual input seen by the model, it is likely that some examples cannot be correctly classified due to missing information in the input.

### Future prediction

The model predicts the future ECG signal based on a present ECG signal. This is a type of unsupervised representation learning, which is our adaptation of Contrastive Predictive Coding^34^ to ECG data. For each ECG frame, we collect a number of consecutive frames, which together are referred to as the context (present). The future is an ECG frame collected at some distance from the context. The distance is measured in the number of frames that lie between the context and the future and we refer to it as the offset. Instead of predicting the future frame directly using a generative model, the context and the future are encoded into vector representations in a way that maximally preserves the mutual information between them. By maximizing the mutual information, the model learns to extract the underlying latent variables, which are shared by the context and the future. This is achieved by optimizing a loss based on Noise Contrastive Estimation^36^. Given an encoding of the context and encodings of many potential future frames, the model predicts which future frame is correct, i.e. it picks the positive sample among many negative samples, which are sampled from the data set. Intuitively, when predicting further into the future, the model learns to infer more global structure shared by the context and the future frame. At the same time, the model discards low-level information and noise that is more local.

### Finetuning

We finetune the pretrained CNNs on the PhysioNet/CinC Challenge 2017 data set, which was collected to encourage the development of methods to classify heart arrhythmia from short ECG recordings. The data set consists of 8528 labeled episodes which were recorded by AliveCor devices. Each recording contains a short (9–60 s) single ECG lead sampled at 300 Hz that belongs to one of the following classes: AF, Normal, Other or Noise (too noisy to classify).

We preprocess each recording by first standardizing the signal using mean and standard deviation computed over the entire data set, then downsampling the signal from 300 to 250 Hz to match the sampling frequency of Icentia11K. Additionally, we pad some recordings with zeros in order to have an uniform signal length of 60 s. Before training, we randomly split the data set into train, test and validation sets at 75%, 20% and 5% of recordings respectively, while maintaining the class ratio in each set. Note that we do not use the hidden test set from the challenge as it remains inaccessible to the public.

Before finetuning a CNN, we replace its output layer (i.e. classification layer) with a fully connected layer whose weights are randomly initialized and whose outputs match the classes of the PhysioNet/CinC Challenge 2017 data set. The CNNs are trained end-to-end (we do not freeze any pretrained weights) for up to 200 epochs. If the training accuracy does not improve for 50 epochs, the training is interrupted. Usually, the network achieves near 100% accuracy on the train set in a short time. As a consequence, the actual number of epochs is less than 200.

During finetuning, we record the macro $F_1$ score, which is used as a metric in the PhysioNet/CinC Challenge 2017, on the validation set after each epoch. After the training is finished, we revert the weights of the network to the checkpoint at which the model had the highest macro $F_1$ score on the validation set. Finally, we record the macro $F_1$ score achieved by the finetuned CNN on the test set.

In order to evaluate a pretraining method, we repeat the described finetuning procedure 10 times. Each time, we draw new train and validation sets from the pool of 80% recordings, adjust the output layer, finetune the model and finally record the macro $F_1$ score on the test set. We measure the performance of a pretraining method based on the statistics gathered from these 10 runs. Specifically, we report the average of macro $F_1$ scores on the test set after 10 runs as well as the standard deviation.

### The model architecture

Throughout this work, we use residual networks^21^ for ECG classification. In the following paragraphs, we describe the architecture of residual networks and the architecture of our future prediction framework.

### Convolutional neural network

We use ResNet-18v2^22^ as the baseline CNN. Architecturally, our networks follow the design of the improved residual networks proposed by He et al.^22^ with some small adjustments. Due to the dimensionality of the input (i.e. ECG data is 1-dimensional), we replace the 2-dimensional convolutional layers with their 1-dimensional counterparts. Furthermore, we use larger filter sizes, i.e. 7, 5, 5, and 3 at each stage respectively, which we have observed to outperform the suggested smaller $3\times 3$ filters. The remaining architectural decisions and hyperparameters, including the weight initialization scheme, are the same as in He et al.^22^.

### Future prediction framework

The framework for Contrastive Predictive Coding^34^ consists of two models that are trained jointly: an encoder model that encodes the ECG frames and an autoregressive model that summarizes the encoded context frames into a single vector representation of the context (Fig. 2). For the encoder **E**, we use the ResNet-18v2 described above. Since we are interested in extracting features from the ECG frames, rather than classifying them, we remove the output (classification) layer from the model, leaving global average pooling as the final layer. We denote the encoded future frames as feature vectors $h_i$. The encoded context frames are passed to an attention pooling^37^ module, which represents the autoregressive model. Attention pooling consists of Transformer^23^ layers with the self-attention mechanism. The module receives the encoded context frames preceded by a learnable vector $c_0$ that represents the token embedding of the pooled context. We use the output corresponding to the input $c_0$ as the pooled context vector *c* and discard the remaining outputs. By using the Transformer as a pooling operation, we allow the model to learn the type of pooling that is best suited for the task, as opposed to using a predefined operation, e.g. max pooling or average pooling. The resulting context vector *c* is used in a dot product to compute the similarity with each encoded future frame $h_i$. Finally, the similarity scores are passed to a softmax layer that allows the outputs of the framework to be interpreted as the probability of a future frame being the positive sample.

The entire framework is trained end-to-end with gradients backpropagated from the cross-entropy loss of classifying the positive sample correctly. Architecturally, the Transformer layers in the attention pooling module are consistent with the Encoder layers proposed by Vaswani et al.^23^ Similar to the attention pooling in Trinh et al.^37^, we decrease the size of the original Transformer by using the following hyperparameters: $N=3$ Transformer layers, $d_{model}=512$ model dimensionality, $h=8$ attention heads, $d_{ff}=2 \cdot d_{model}=1024$ inner dimensionality and no dropout (refer to Vaswani et al.^23^ for more information on the hyperparameters). Compared to other pretraining methods, the future prediction framework requires more parameters to be trained. Furthermore, due to the way this task is structured, the residual network learns from more ECG frames in a single pass. Consequently, we observe longer training times for this type of pretraining.

### Training

Since we are able to represent every task as a classification problem, in each task we minimize the categorical cross-entropy loss. All models are trained with Adam^38^ optimizer that is initialized with the default hyperparameters. Furthermore, we use different batch sizes depending on the number of trainable parameters and the available memory on the GPU cards. As a rule of thumb, we try to pick a batch size that allows us to use as much of the GPU memory as possible. All models are trained on Nvidia Tesla P100 GPUs.

## Experiments and results

We now analyze the effectiveness of pretraining with respect to the performance on the downstream task. In other words, we measure how much does the pretraining improve the macro $F_1$ score on our test set for the PhysioNet/CinC Challenge 2017. To that end, we conduct a series of experiments that test the efficacy of pretraining in different settings. We compare the pretraining methods with random weight initialization, which we consider as the baseline method. First, we investigate several configurations (i.e. a set of hyperparameters) of each pretraining method to determine which configuration is best suited for the downstream task. Further, we explore how the size of the downstream data set affects the performance of pretraining methods. Next, we change the sampling frequency of the downstream data set to evaluate how well the pretrained networks generalize to ECG data with different properties. In a further experiment, we investigate how changing the depth of the residual network affects the performance of pretraining methods. Finally, we explore how well the pretrained networks perform on other downstream data sets.

### Hyperparameters of pretraining methods

The hyperparameters of pretraining methods are parameters that control the pretraining process. They determine the shape of the input (e.g. the size of ECG frame) and the properties of the task (e.g. the offset or the number of negative samples control the difficulty of the future prediction task), thus they have an indirect impact on what features the network will learn to extract. We first investigate different sets of hyperparameters of each pretraining task, which we refer to as configurations, in order to discover which configurations perform well on the downstream task, i.e. AF classification.

Table 1 compares different configurations of the pretraining methods. In the classification tasks, we explore 3 different sizes of ECG frames. In the future prediction task, we examine different combinations of the context size, number of negative samples and offset. We compare the configurations based on the average macro $F_1$ score (abbreviated as $F_1$) on the downstream test set. Additionally, we report the average of $F_1$ scores for each class (abbreviated as $F_{1x}$ where *x* is a class identifier). The results show that all pretraining methods outperform random weight initialization in predicting every class. Looking at the best configuration of each pretraining method in terms of $F_1$, the improvement over the baseline (i.e. random weight initialization) is 6.57% for beat classification, 4.92% for rhythm classification, 4.79% for heart rate classification and 3.69% for future prediction. When it comes to the size of ECG frame, the preferred frame is about 8 s long for beat and rhythm classification, and about 2 s for the heart rate classification. We suspect that during finetuning, the network focuses more on local patterns over long-term dependencies in the data. The small frame size is especially interesting in case of the heart rate pretraining due to the way how the labels are generated. Recall that for a frame size of about 2 s we generate the label from a 4 s window centered on that frame. Therefore, the network must cope with a lot of missing information when classifying the frames. Regarding future prediction, increasing the difficulty of the task by setting a larger offset and adding more negative samples seems to produce inconclusive results with respect to any improvement of performance. For the remainder of the paper, when discussing a particular pretraining method, we will be referring to its best configuration.

**Table 1:** Comparison of different configurations of the pretraining methods.

Pretraining method				Frame	$F_1$	$F_{1n}$	$F_{1a}$	$F_{1o}$	$F_{1p}$
None (random weight initialization)					.731 (± .019)	.898 (± .005)	.711 (± .027)	.701 (± .017)	.613 (± .062)
Beat classification				512	.769 (± .011)	.911 (± .010)	.760 (± .018)	.758 (± .016)	.647 (± .022)
				2048	**.779 (**± **.014)**	**.915 (**± **.007)**	**.777 (**± **.014)**	**.763 (**± **.014)**	**.661 (**± **.040)**
				4096	.768 (± .010)	.908 (± .009)	.764 (± .021)	.754 (± .015)	.646 (± .025)
Rhythm classification				512	.742 (± .017)	.896 (± .007)	.721 (± .026)	.716 (± .032)	.636 (± .045)
				2048	.767 (± .012)	.908 (± .004)	.753 (± .020)	.745 (± .018)	.660 (± .026)
				4096	.755 (± .005)	.903 (± .008)	.745 (± .022)	.735 (± .012)	.635 (± .017)
Heart rate classification				512	.766 (± .011)	**.915 (**± **.004)**	.759 (± .019)	.756 (± .015)	.635 (± .029)
				2048	.753 (± .013)	.910 (± .005)	.743 (± .037)	.738 (± .011)	.619 (± .039)
				4096	.751 (± .010)	.909 (± .006)	.744 (± .019)	.739 (± .016)	.611 (± .025)

	Context	ns	Offset	Frame					
Future prediction	8	4	2	512	.756 (± .008)	.903 (± .007)	.742 (± .011)	.730 (± .017)	.649 (± .021)
	16	8	2	512	.744 (± .016)	.905 (± .005)	.730 (± .027)	.730 (± .009)	.612 (± .041)
	16	8	8	512	.758 (± .013)	.908 (± .005)	.753 (± .021)	.745 (± .012)	.627 (± .026)
	16	16	8	512	.745 (± .013)	.897 (± .006)	.724 (± .024)	.722 (± .009)	.639 (± .034)

For each method, we report the average macro $F_1$ score (and the standard deviation) on our test set for the PhysioNet/CinC Challenge 2017^7,8^. Additionally, we report the average $F_1$ score for each class: normal ($F_{1n}$), AF ($F_{1a}$), other ($F_{1o}$) and noisy ($F_{1p}$). *Frame* refers to the length of an ECG frame, *context* to the number of frames in the context, *ns* to the number of negative samples and *offset* to the distance between the context and the future frame measured in frames. All pretraining methods outperform random weight initialization in predicting every class.

**Table 2:** Comparison of the pretraining methods depending on the size of the downstream train set.

Pretraining method	25% train	50% train	75% train
None (random weight initialization)	.670 (± .013)	.712 (± .010)	.731 (± .019)
Beat classification	**.739 (**± **.014)**	**.763 (**± **.011)**	**.779 (**± **.014)**
Rhythm classification	.707 (± .018)	.727 (± .028)	.767 (± .012)
Heart rate classification	.722 (± .010)	.749 (± .018)	.766 (± .011)
Future prediction	.694 (± .014)	.734 (± .011)	.758 (± .013)

For each method, we report the average macro $F_1$ score (and the standard deviation) on our test set for the PhysioNet/CinC Challenge 2017^7,8^. We examine 3 sizes of the train set as a proportion of the entire data set: 25%, 50% and 75% (original split). Pretraining allows models to be trained on less data and still achieve the same degree of performance as the same models that are not pretrained.

**Table 3:** Comparison of the pretraining methods depending on the sampling frequency (Hz) of the downstream data set.

Pretraining method	128 Hz	250 Hz (Icentia11K)	300 Hz (PhysioNet)
None (random weight initialization)	.701 (± .017)	.731 (± .019)	.715 (± .023)
Beat classification	**.779 (**± **.012)**	**.779 (**± **.014)**	**.770 (**± **.011)**
Rhythm classification	.748 (± .012)	.767 (± .012)	.747 (± .017)
Heart rate classification	.761 (± .011)	.766 (± .011)	.767 (± .010)
Future prediction	.747 (± .008)	.758 (± .013)	.734 (± .016)

For each method, we report the average macro $F_1$ score (and the standard deviation) on our test set for the PhysioNet/CinC Challenge 2017^7,8^. Note that all networks are pretrained on ECG data sampled at 250 Hz, regardless of the sampling frequency during finetuning. Pretraining is beneficial even if networks are not specifically trained to deal with ECG data sampled at different frequencies.

We now take a closer look at the validation performance in terms of $F_1$ of each pretraining method during finetuning (Fig. 3). Pretrained networks achieve a high validation $F_1$ within just several epochs, whereas their randomly initialized counterparts take longer to converge to a stable validation $F_1$. Furthermore, pretrained networks consistently show better validation performance than randomly initialized networks over the course of finetuning. Evidently, pretraining not only improves the performance, but also accelerates the training.

### Size of the downstream data set

So far we evaluated our pretraining methods on a medium sized (n $=$ 8528) ECG data set. Now, we investigate the extent of pretraining’s effectiveness when finetuning on data sets of different sizes. To that end, we finetune the networks only on a subset of the PhysioNet/CinC Challenge 2017 data set. Recall that the original data split was: 75% train, 5% validation and 20% test. In the following experiment, we maintain the test and validation sets, and only reduce the size of the train set to 50% and 25% of the entire data set.

Table 2 reports the average macro $F_1$ score of each pretraining method on the downstream test set, depending on the size of the downstream train set. For beat and heart rate classification, the performance improvement over the baseline (i.e. random weight initialization) grows when the data set size decreases. For rhythm classification and future prediction, the performance improvement remains similar across various data set sizes. The narrowing of the performance gap could suggest that once the data set is big enough, pretraining becomes redundant. Notably, if a residual network pretrained for beat classification is finetuned on only 25% of train data, it still performs better than its randomly initialized counterpart trained on 75% of data. This shows that pretraining allows models to be trained on less data and still achieve the same degree of performance as the same models that are not pretrained.

### Sampling frequency of ECG signal

ECG databases vary in the characteristics of the ECG signal such as length, number of leads, sampling frequency, or processing artefacts. These differences arise mostly from the properties of the device that records the ECG. Therefore, transfer learning approaches for ECG data should ideally be unaffected by the variations between signals. In this section, we investigate how well the pretrained networks perform on ECG data sampled at a frequency different than during pretraining. This is of particular interest to us, because we reduce the sampling frequency of the downstream data set to match that of the upstream data set. This results in a data loss that could lead to a degradation of performance. For this reason, by trying different sampling frequencies, we want to eliminate the possibility that downsampling causes the performance to decrease more than the pretraining does to increase it. Note that we maintain the same relative input length, i.e. about 60 s, across different sampling frequencies, zero-padding the input where necessary.

Table 3 reports the average macro $F_1$ score of each pretraining method on the downstream test set, depending on the sampling frequency of ECG data. Besides finetuning the models on the train set using the original sampling frequency (other preprocessing methods are still applied), we also measure the performance of our methods when the sampling frequency of the downstream data set is almost 2 times smaller than the frequency the networks were pretrained on (128 Hz vs 250 Hz). As expected, we observe a performance decline when the sampling frequency changes. However, all pretraining methods outpeform random weight initialization no matter the sampling frequency. Pretraining is beneficial even if networks are not specifically trained to deal with ECG signals sampled at different frequencies. Interestingly, randomly initialized networks actually perform worse ($-\,2.19\%$ $F_1$) on the original data set, which could be connected to the increased input dimensionality.

### Architecture of the residual network

Due to the ever-increasing amounts of data and computing power that are available for training, deep learning models have grown in size to accommodate more knowledge and improve the performance. In light of this trend, ResNet-18v2, which is our baseline model, can be considered a “shallow” network. Compared to other residual networks, ResNet-18v2 has a small number of layers and parameters. Therefore, we now increase the size of the model. Specifically, we employ two new architectures: ResNet-34v2^22^ and ResNet-50v2^22^, which replaces standard residual blocks with bottleneck blocks. Other hyperparameters of the network remain the same. Notably, in the future prediction task, we pretrain only the first 3 stages of the deeper ResNet-50v2 due to a high model complexity. Since the bottleneck architecture increases the number of output channels by 4 times, the dimensionality of the attention pooling module (i.e. $d_{model}$) increases by the same amount, which leads to a significant increase in the number of trainable parameters.

**Table 4:** Comparison of the pretraining methods depending on the architecture of the model (i.e. residual network).

Pretraining method	ResNet-18v2	ResNet-34v2	ResNet-50v2
None (random weight initialization)	.731 (± .019)	.764 (± .012)	.708 (± .023)
Beat classification	**.779 (**± **.014)**	**.794 (**± **.018)**	**.775 (**± **.015)**
Rhythm classification	.767 (± .012)	.775 (± .020)	.760 (± .008)
Heart rate classification	.766 (± .011)	.771 (± .008)	.761 (± .019)
Future prediction	.758 (± .013)	.761 (± .014)	.743* (± .010)

For each method, we report the average macro $F_1$ score (and the standard deviation) on our test set for the PhysioNet/CinC Challenge 2017^7,8^. Employing the ResNet-34v2 improves the performance of every pretraining method. We suspect that ResNet-34v2 lies in a sweet spot between model complexity and performance, whereas ResNet-18v2 underfits and ResNet-50v2 overfits to the training data.

*Due to a spike in the model complexity, we only pretrain the first 3 stages of the ResNet-50v2.

**Table 5:** Comparison of the pretraining methods on two new downstream data sets.

	PTB-XL^8,28^		ICBEB2018^39^			
	AUC	$F_{max}$	AUC	$F_{max}$	$F_{\beta =2}$	$G_{\beta =2}$
**Pretraining method**						
None (random weight initialization)	.942 (± .016)	.918 (± .004)	.954 (± .008)	.833 (± .017)	.787 (± .025)	.553 (± .028)
Beat classification	.962 (± .006)	**.926** (± .004)	.961 (± .004)	.854 (± .005)	.814 (± .008)	.591 (± .012)
Rhythm classification	.958 (± .013)	.922 (± .006)	.956 (± .006)	.848 (± .012)	.807 (± .016)	.575 (± .023)
Heart rate classification	**.965 (**± **.003)**	.923 (± .003)	.951 (± .006)	.833 (± .012)	.790 (± .013)	.558 (± .018)
Future prediction	.955 (± .004)	.920 (± .003)	.955 (± .005)	.844 (± .011)	.802 (± .017)	.572 (± .021)
**Related work**						
Strodthoff et al.^28^	.957 (± .015)	.917 (± .008)	**.974 (± .005)**	**.855 (± .020)**	**.819 (± .028)**	**.602 (± .044)**

For each method, we report the average performance (and the standard deviation) on the respective test sets. We observe performance improvements from pretraining on both data sets.

Bold numbers in ICBEB2018 dataset reflect the best overall performance (i.e. that of Strodthoff et al.^28^), instead of the best pretraining performance (i.e. Beat classification).

Table 4 reports the average macro $F_1$ score of each pretraining method on the downstream test set, depending on the architecture of the model. Employing ResNet-34v2 improves the performance of every pretraining method: 4.51% increase for random weight initialization, 1.93% for beat classification, 1.04% for rhythm classification, 0.65% for heart rate classification and 0.40% for future prediction. However, further increase in the complexity from using ResNet-50v2 leads to a decline in performance. The decline is much more steep in case of no pretraining: − 7.33% for random weight initialization versus − 2.39% for beat classification. We suspect that ResNet-34v2 lies in a sweet spot between model complexity and performance, whereas ResNet-18v2 underfits and ResNet-50v2 overfits to the training data. It should be noted that while the number of trainable parameters increased, the number of pretraining steps remained the same. During pretraining, we recorded lower validation performance for the deeper models, which leads us to believe that we may have finished pretraining too soon. This was particularly noticeable in case of future prediction, whose model complexity is higher than any other method.

### Downstream data set

A desirable property of pretrained networks is that they can be finetuned to not one but a number of related tasks. Therefore, we now investigate how well our pretraining methods apply to different downstream (target) data sets, i.e. how generalizable the pretrained feature extractors are. To that end, we choose two new downstream data sets: the PTB-XL database^8,28^ and a data set released for the 1st China Physiological Signal Challenge 2018 held during the 7th International Conference on Biomedical Engineering and Biotechnology (ICBEB 2018)^39^. The ICBEB2018 data set is also a part of the training data in the PhysioNet/CinC Challenge 2020^8,40^.

The PTB-XL database contains 21,837 12-lead ECG recordings that were sampled at 500 Hz and last exactly 10 s. Further, there are 71 different statements, which are used as annotations. They are assigned to three categories: diagnostic, rhythm and form. Here, we only use the rhythm annotations. Consequently, we select only the recordings that have at least one rhythm label, effectively shrinking the size of the data set to 21,066 recordings. Further, we use the recommended 10-fold train-test split, i.e. we use folds 1–8 as the train set, fold 9 as the validation set and fold 10 as the test set. Finally, we standardize the recordings using mean and standard deviation computed over the entire database, and downsample the recordings to 250 Hz.

The ICBEB2018 data set contains 6877 12-lead ECG recordings that were sampled at 500 Hz and last 6–60 s. Each recording has up to three annotations that describe a normal sinus rhythm or a heart condition. Since the original test set is kept private, we reserve 20% of recordings for testing and split the remaining 80% in train (75%) and validation (5%) sets, maintaining the original class ratio in each set. Similarly to the PTB-XL database, we standardize the recordings, downsample them to 250 Hz and pad with zeros to maintain an uniform signal length of 60 s.

In contrast to the PhysioNet/CinC Challenge 2017 data set used so far, which contains 1-lead ECG recordings, the aforementioned new data sets comprise 12-lead ECG recordings. As a consequence, the pretrained models, which also expect a single lead ECG recording, must be adjusted to accommodate this new data format. Recall that residual networks begin with a convolutional layer that expects a variable-length input signal with a fixed number of input channels, in our case one channel. In order to increase the number of input channels, while maintaining the same output dimensions, we simply duplicate the learned filters for each additional channel and scale all weights in the layer by multiplying them by the ratio of the original to adjusted input channels, in our case $\frac{1}{12}$. In doing so, we hope that the learned feature extractors will generalize to other ECG channels.

We use the pretrained ResNet-34v2, and similarly to the previous experiments, we first finetune each model 10 times, then average the performance on the test set. In case of the ICBEB2018 data set, we additionally resample the train and validation set from the pool of 80% recordings before finetuning a model. Since we deal with multi-label classification tasks (i.e. multiple labels can be assigned to a single instance), we change the activation function of the output of residual network to sigmoid and train the model with binary cross-entropy loss. When testing, we use model weights from the epoch where the model achieved best validation loss.

We evaluate the performance of our pretrained methods on the respective test sets using 4 new metrics. Following Strodthoff et al.^28^, we compute the averaged class-wise *AUC* (abbreviated as *AUC*) and a sample-centric $F_{max}$ that summarizes a threshold dependent $F_1$ score by single number, which is the maximum $F_1$ score found by varying the decision threshold. Further, we employ two metrics used in the PhysioNet/CinC Challenge 2020: a general class-weighted F-score $F_{\beta =2}$ and a generalization of the Jaccard measure $G_{\beta =2}$. We convert the output probabilities to binary decision using a threshold found independently for each metric on the train set.

Table 5 reports the average performance of each pretraining method on the two new downstream test sets. Since the ranking of methods is the same independent of the performance metric, we focus on *AUC* when presenting the results. We note performance improvements on both data sets from the pretraining. For PTB-XL, the relative increase in *AUC* compared to the baseline (i.e. random weight initialization) is $2.12\%$ for beat classification, $1.70\%$ for rhythm classification, $2.44\%$ for heart rate classification and $1.38\%$ for future prediction. For ICBEB2018, we observe a smaller performance improvement and even a decline in case of heart rate classification: $0.73\%$ for beat classification, $0.21\%$ for rhythm classification, $-\,0.31\%$ for heart rate classification and $0.10\%$ for future prediction.

## Discussion

Transfer learning proves to be a valuable technique to deal with an inadequate amount of annotated data that plagues models trained to classify ECG recordings. In this work, we showed that pretraining convolutional neural networks (CNN) on a large ECG database and subsequently finetuning them on a much smaller ECG data set considerably improves the performance on the target task, effectively reducing the number of expensive annotations required to achieve the same performance level as CNNs that are not pretrained. We suspect that the additional data diversity experienced during the pretraining stage contributes to networks’ ability to generalize to unseen data after the finetuning stage. Furthermore, we conducted a series of experiments that test the robustness of pretraining. Specifically, we used the pretraining methods on: different models (various residual network architectures), different qualities of ECG data (simulated by changing the sampling frequency), different data set sizes and finally different downstream data sets. In all experiments, the pretrained networks performed better on the target task than the randomly initialized networks, as shown by several performance metrics. We believe that collecting a massive ECG database that reflects the heterogeneity of ECG data would facilitate the pretraining of large networks that can be finetuned to a variety of ECG related tasks, much like CNNs pretrained on ImageNet are often used in various computer vision tasks.

ECG annotations are often expensive to acquire, which limits the size of many ECG data sets. For this reason, we also explored unsupervised and self-supervised (i.e. labels are generated automatically) pretraining in the ECG domain. Similar to the more common supervised pretraining, CNNs pretrained in an unsupervised and self-supervised manner perform better than CNNs that were not pretrained and sometimes they even match the performance of supervised pretraining. Nonetheless, supervised pretraining remains a better choice if the labels are available.

The performance of pretraining methods depends on how the pretraining task is modelled as evidenced by the small hyperparameter study. Intuitively, the way a pretraining task is defined and modelled has a direct impact on what the model learns, such that if the upstream and downstream tasks do not share many commonalities, then the pretrained feature extractors may turn out to not be of much use for the downstream task. Notably, this also applies to the way we choose the labels in the classification tasks, which is a part of the task definition that we have not investigated in this work.

Lastly, we want to briefly address some difficulties that we have encountered and insights related to the pretraining. We have pretrained the CNNs on just a small fraction of the upstream data set, i.e. less than 20%. Furthermore, we did not observe plateauing loss, which leads us to believe that we could have pretrained longer. Further, we noticed that certain configurations of the future prediction framework fail to converge. This occurred when the task became too difficult due to a large offset or a large number of negative samples. We think that future research should be directed towards studying different techniques for unsupervised or self-supervised pretraining, because these methods do not rely on human annotations that can be very expensive to acquire for ECG data.

## Conclusion

In this work, we used transfer learning to improve convolutional neural networks (CNN) trained to classify heart rhythm from a short ECG recording. First, we pretrained CNNs on a large data set of continuous raw ECG signals. Next, we finetuned the networks on a small data set for classification of Atrial Fibrillation (AF). We showed that pretraining CNNs improves the performance on the target task, i.e. AF classification, by up to $6.57\%$, effectively reducing the number of annotations required to achieve the same performance as CNNs that are not pretrained. Furthermore, we showed that unsupervised pretraining on ECG data is a viable method for improving the performance on the target task, albeit to a lesser extent than supervised pretraining. Nonetheless, we believe that unsupervised pretraining will become more relevant since it does not rely on annotations, which are expensive to acquire for ECG data.
